# Household income differences in food sources and food items purchased

**DOI:** 10.1186/1479-5868-7-77

**Published:** 2010-10-26

**Authors:** Simone A French, Melanie Wall, Nathan R Mitchell

**Affiliations:** 1 Division of Epidemiology & Community Health, School of Public Health, University of Minnesota, Minneapolis, Minnesota, USA; 2 Division of Biostatistics, School of Public Health, University of Minnesota, Minneapolis, Minnesota, USA; 3 Division of Epidemiology & Community Health, 1300 South 2nd St, Suite 300 Minneapolis, Minnesota 55454, USA

## Abstract

**Background:**

The present study examined income-related household food purchases among a sample of 90 households from the community.

**Methods:**

Annotated food purchase receipts were collected for a four-week period by the primary household shopper. Receipt food source and foods items were classified into specific categories, and food quantities in ounces were recorded by research staff. For home sources, a limited number of food/beverage categories were recorded. For eating out sources, all food/beverage items were recorded. Median monthly per person dollars spent and per person ounces purchased were computed. Food sources and food categories were examined by household income tertile.

**Subjects and Setting:**

A community-based sample of 90 households.

**Results:**

Higher income households spent significantly more dollars per person per month from both home and eating out sources compared with lower income households ($163 versus $100, p < .001). Compared with lower income households, higher income households spent significantly more home source dollars on both fruits/vegetables (21.5 versus 10.2, p < .001) and sweets/snacks (17.3 versus 8.3, p < .001), but did not differ on home dollars spent on sugar sweetened beverages (2.0 versus 1.7, p < .46). The proportion of home beverages that were sugar sweetened beverages was significantly higher among lower income households (45% versus 26%, p < .01). Within eating out sources, lower income households spent a significantly greater percent of dollars per person at carry out places (54% versus 37%, p < .01). No income differences were observed for dollars spent at discount grocery stores, small grocery stores or convenience stores.

**Conclusions:**

Higher income households spent more money on both healthy and less healthy foods from a wide range of sources. Lower income households spent a larger proportion of their eating out dollars at carry out places, and a larger proportion of their home beverage purchases were sugar sweetened beverages.

## Introduction

### Lower income is associated with a poorer diet

Population-based surveys of individual intake show that lower income is associated with a poorer quality diet [1]. Individuals with lower income consume fewer fruits and vegetables, a greater proportion of energy from fat, and less fiber compared to higher income individuals [1]. Data show that income disparities have a greater effect on dietary quality rather than on amount of calories consumed [1-3].

### Household food purchases and individual dietary intake quality

Examining food purchase patterns at the household level may provide insight into potential reasons for less healthful individual food intake. Household food purchases may influence individual intake through simple availability and from social influences such as behavioral modeling [4]. Lower income households purchase fewer fruits and vegetables compared with higher income households [5] and are more likely to patronize fast food restaurants than full-service restaurants when eating out [6].

Two possible reasons for these differences in foods purchased and food sources selected are related to food cost and food availability. When food cost constraints are high, as they are among lower income households, shoppers may choose more energy dense foods to maximize the calories obtained per dollar expended [1,3,7,8]. This may result in less purchase of nutrient dense foods such as fruits and vegetables or cheaper versions of certain food items [7,9]. The poor may also be constrained in the availability of food stores that are accessible to them. For example, lower income households may live in neighborhoods that lack large discount chain supermarkets and thus may shop for food at higher-priced small grocery or convenience stores [10-12].

Eating out is another possible means by which household income may influence individual dietary quality [13,14]. About half of U.S. food dollars are spent at eating out places [15,16]. Lower income households may choose less expensive restaurants when eating out, such as fast food places [6]. These eating out sources may offer less healthful, energy dense, but cheaper, foods [17].

The household food purchase receipt or record is a method that has been used to examine household food purchases (for a review, see French et al. [4,18-27]). Household food purchase receipt and record studies have focused on grocery store food purchases in relation to individual dietary intake. Most of these studies focused on nutrients and energy, and not on purchase and consumption at the food level. No studies have examined household food purchase receipts from eating out sources, despite the fact that eating out sources comprise about half of the US household food expenditures [13-16]. Most of these studies find modest agreement between household grocery store purchases and individual dietary intake [4,18-27].

The purpose of the present research was to explore income-related differences in household level food purchases that might be influenced by access to food sources and by food costs. The present paper reports associations between household income, food sources and food purchases among a community-based sample of 90 households. It was hypothesized that lower income households would be more likely than higher income households to spend their food dollars on home sources, such as grocery stores, rather than eating out sources. However, among eating out sources, it was hypothesized that lower income households would be more likely to spend food dollars at carry out places compared to full-service restaurants. Lower income households were hypothesized to spend more of their home source dollars at small grocery stores, convenience stores and gas stations, compared with higher income households. Lower income households were hypothesized to purchase fewer fruits and vegetables, more sweets and snacks and more sugar sweetened beverages compared to higher income households.

## Experimental Methods

### Study Population and Recruitment

Data for the present study were collected as part of a community-based household weight gain prevention intervention conducted in Minneapolis, Minnesota, USA in 2007-2008 (French SA, Gerlach A, Mitchell N *et al*. Household obesity prevention intervention targeting television, soft drinks, fast food and physical activity (*under review*). The study was a group-randomized trial, with households the unit of randomization, intervention and analysis. Data presented here are cross-sectional baseline data only. Households were recruited from the community over an eight-month period. Recruitment sources included community libraries, worksites, schools, daycare centers, health clinics, religious institutions, park and recreation centers, grocery stores and food co-ops. Fliers and brochures were distributed in local community centers, libraries, grocery stores and schools. In-person recruitment was conducted at community events, health fairs, and after-school programs. Interested households contacted study staff and were screened for eligibility. Study eligibility criteria were related to the weight gain prevention intervention aims. Eligibility criteria included: 1) at least one child ages ≥5 yrs and two household members ages ≥12 yrs; 2) residence in a private house or apartment within 20 miles of the university; 3) household TV viewing weekly average of ≥10 hrs per person; 4) no household members with dietary, medical, psychological, or physical limitations that would prevent their participation in intervention activities; and 5) willingness to be randomized to active intervention or control group. Eligibility was assessed during an initial telephone call conducted by a trained staff member and confirmed during an in-person baseline clinic visit. Overall, 732 inquiries were received, 289 families were eligible, 106 households completed the baseline clinic visit, and 90 households completed the baseline home visit and 4 week receipt data collection and thus were enrolled in the study and randomized. The University of Minnesota IRB approved the study.

### Data Collection Methods

Households completed a face-to-face clinic visit during which all household members completed weight and height measures, and survey self-report measures of eating behaviors and physical activity. Following the clinic visit, a home visit was scheduled. During the home visit, the research staff person trained the primary household shopper on the receipt collection and annotation protocol. The receipt collection training required about 10-15 minutes. A home food inventory was completed by the research staff during the home visit. Home visits required between 45 minutes and 2 hours, depending on the amount of food present in the household.

Primary shoppers were instructed to collect and annotate receipts and mail them to study staff on a weekly basis. Receipt annotation sheets queried the primary shopper for information about the food item, quantities, cost and source (name and type). Primary shoppers were instructed to query other household members about any food purchases they made during the week. The primary shopper was instructed to complete a receipt annotation sheet regardless of whether a receipt was available (e.g., if food was purchased from a vending machine or convenience store and no receipt was provided). For simplicity, we use the term receipt to refer to receipt annotation sheet hereafter.

Due to the complexity and time required for receipt annotation, a decision was made to limit grocery store annotation to foods and beverages targeted by the intervention because of their potential link to excess weight gain. The specific targeted food categories were: fruits, vegetables, prepackaged snacks and sweets, prepackaged entrees, and beverages. For eating out receipts, primary shoppers were instructed to record all food and beverage items on the annotation sheet, including entrees, side orders, and beverages such as alcohol and specialty drinks (e.g., lattes, smoothies, frapuccinos). Details about the receipt data collection and coding protocol are available elsewhere [28].

### Statistical Analysis

Statistical analyses were conducted using the Statistical Analysis System Release 8.2. (SAS Institute Cary, NC, USA) [29]. Food and beverage purchases for all receipt annotation sheets were included in all analyses (n = 2,483 annotation sheets). Summary variables were calculated from the receipt data. Quantity in ounces and expenditures in dollars were summed within food and source categories for each household across the 4-weeks of receipts. To adjust for differences in number of people living in the household, per person dollars and ounces variables were created. Specifically, each household quantity variable (e.g., total dollars spent at home food sources on fruits and vegetables) was divided by the number of persons five years and older living in the household. In addition, to examine different ratios of specific food or source purchases, household percent variables were created taking the total purchases in a specific food or source category and dividing by the total purchases in the broader category (e.g. % eating out dollars spent at carry-out sources = total dollars per person spent at carry-out sources divided by total dollars per person spent eating out).

To examine purchases by household income, tertiles of household income were created based on self-reported household income from the clinic data collection visit. Personal pre-tax income was reported only on the adult survey, and all adult income was summed within the household to generate the household income. Median values for each study variable are presented by household income and statistical tests of increasing or decreasing trends across income were performed using the Jonckheere-Terpstra test, which is a non-parametric test for trend [30]. The household demographic characteristics were self-reported by the primary shopper for the household.

## Results

### Receipt completion rates

One hundred six households completed the initial baseline clinic visit. Of these, 90 households completed four weeks of receipt collection and were enrolled in the study. Households were enrolled and randomized only if the baseline clinic visit, the home visit, and the four-week receipt collection activity were completed. Thus, the drop out rate from clinic visit to receipt collection was 15% (90/106 = 85% completers). A total of 2,483 receipt annotation sheets were returned by the 90 households. Of these, 1,892 (75.2%) included receipts plus annotation sheets, and 591 included annotation sheets only (no receipt). A total of 9,498 food line items were included in the receipt annotation sheets.

Households differed in the number of receipts returned to research staff. The number of receipts returned by a household depends on many factors, including household income and the number of adults and children who reside in the household. The median number of receipts returned by high, medium and low income households was 35, 21, and 19, respectively. Only 10 households returned 10 or fewer total receipts during the four week receipt collection period. Of these 10 households, 1, 2, and 7 were high, medium and low income, respectively. Compared with households that turned in more than 10 receipts, households that turned in 10 or fewer receipts reported lower income (US $44,000 versus US $83,101), had primary shoppers who were less educated (30% advanced education versus 64%), and had a higher body mass index (34.7 kg/m^2 ^versus 29.0 kg/m^2^).

### Household descriptive characteristics

Ninety-three percent of the primary shoppers were female, and 78% were white. Households on average were comprised of four people (1.8 adults (range 1-4) and 2.0 children (range 1-5)). The most common configuration was two adults and two children (51%). The primary shopper was on average 40.8 years (SD = 7.3 yrs), 64% were married or living with their significant other; 25.8% had more than a college degree. Reported household income was distributed as follows: 35% ≤ US $45,000 per year; 30% between US $50,000 and $95,000; and 35% ≥ US $100,000 per year. The average body mass index for the primary shopper was 29.7 kg/m^2 ^(SD = 7.2 kg/m^2^). Household primary shoppers reported eating from a carry-out restaurant 2.3 times and from a sit down restaurant 1.1 times during the preceding week. On average, household primary shoppers reported that the household ate six meals together during the preceding week.

### Household food sources by income

Table 1 shows the household weekly dollars spent per person from each food by income group. Overall, higher income households spent more per person from both home and eating out sources compared with lower income households. As a percent of total food spending, lower income households spent 73% of their food dollars from home sources compared with 63% among higher income households (*P *= .09). As a percent of eating out dollars, lower income households spent more at carry out restaurants (54%) compared with higher income households (37%) (*P *= .01).

**Table 1:** Per Person Dollars Spent Monthly from Home and Eating Out Sources by Income*

**Source**	**N Receipts**	**Low**	**Med**	**High**	**P**
Home Sources $	1111	80.06	80.09	105.02	.013
Eating Out Sources $	1372	22.77	36.2	55.4	.002
Total $		100.2	115.44	163.48	.001
% of Total $ from Home Sources		73	71	63	.086
% of Total $ from Eating out Sources		27	29	37	.086
**Eating out Sources**	**N Receipts**	**Low**	**Med**	**High**	**P**
Carry out	756	14.17	12.67	20.34	.055
Restaurants	345	5.93	11.52	27.09	.004
Cafeteria (means)	135	0.93	2.18	3.6	.049
Vending (means)	51	0.12	0.11	0.54	.011
Bar/tavern (means)	15	0.79	0.64	1.02	.227
Other eating out (means)	70	3.25	2.71	3.29	.199
% of eating out $ from carry out		54	49	37	.011
**Home Sources**	**N Receipts**	**Low**	**Med**	**High**	**P**
Discount grocery chain	465	48.88	57.59	52.24	.455
Premium grocery chain (means)	163	5.48	26.90	26.16	.037
Wholesale store (means)	48	4.38	2.85	11.34	.024
Farmers market (means)	17	0.41	0.45	1.17	.094
Small grocery (means)	22	0.17	0.36	0.87	.110
Ethnic market (means)	4	0.04	0.16	0.27	.261
Specialty foods (means)	55	1.39	2.06	4.08	.049
Convenience store	199	3.91	2.90	4.25	.239
Gas station (means)	67	0.97	0.49	1.26	.095
Non-food store (means)	43	0.47	1.11	0.31	.296
Unknown store (means)	26	0.26	0.13	0.73	.327
Mail order	2	---	---	---	---
% of home $ from convenience & gas store		10	10	7	.425

*Medians presented unless otherwise indicated by (means). Means are presented when medians are equal to zero due to lower frequency of particular purchase source.

Household per person monthly dollars spent was significantly higher among middle and high income households for premium chain grocery stores, and among high income households for wholesale stores and specialty food stores. No significant income differences were observed for per person dollars spent at discount chain grocery stores, small grocery stores, convenience stores, gas stations or nonfood stores.

### Household foods purchased by income

Table 2 shows the household monthly per person dollars spent for each food category by household income group. Dollars spent at home sources (left panel) and eating out sources (right panel) are presented. Compared with lower income households, home and eating out fruit and vegetable purchases (dollars per person), home purchases for snacks and sweets, and eating out entrees and side order purchases were significantly higher among high income households.

**Table 2:** Per Person Dollars Monthly for Food Categories from Home and Eating Out Sources by Income

	Home Monthly Dollars Spent* by Income				Eating Out Dollars Spent* by Income			
**Food Category**	**Low**	**Med**	**High**	**P**	**Low**	**Med**	**High**	**P**

Foods								
Fruits/vegetables (includes potatoes)	10.27	16.65	21.56	.001	1.39	1.75	3.17	.003
Sweets/snacks	8.38	15.77	17.36	.001	1.13	2.10	2.45	.061
Prepackaged entrée								
<500 cal	0.92	1.35	0.92	.481	--	---	---	---
>500 cal	2.88	3.20	2.08	.375	---	---	---	---
Eating out entrée	---	---	---	---	15.59	19.61	33.1	.003
Eating out side	---	---	---	---	0.0	0.68	0.99	.014
								
Beverages								
Sugar beverages	1.75	2.67	2.08	.464	1.15	1.23	1.75	.056
Non-caloric beverages	0.0	0.87	3.12	.001	0	0	0.82	.001
Fruit/vegetable juice	1.34	1.54	1.66	.091	0	0	0	.220
Sum of all home (or away) beverages	4.12	6.56	9.31	.014	3.33	5.48	10.49	.002
Eating out alcoholic beverages (means)	---	---	---	---	1.54	1.77	3.31	.029
Eating out specialty drinks (means)	---	---	---	---	1.41	2.93	3.61	.012
% of home $ from sweets/snacks	14	18	19	.009	---	---	---	---
% of home $ from fruits/vegetables	14	16	22	.001	---	---	---	---
% home beverage $ from sugar beverage	45	38	26	.014	---	---	---	---
Ratio of sweets/snacks $ to fruits/vegetables $	0.95	0.89	0.97	.473	---	---	---	---

* Median dollars per person unless noted by (means)

No income-related differences were observed for sugar sweetened beverage purchases from home or eating out sources. However, the percent of home beverage dollars spent for sugar sweetened beverages was significantly higher among lower income households (45%) compared with higher income households (26%). Higher income households spent significantly more dollars per person on non-caloric beverages from both home and eating out sources. The relative proportion of per person dollars spent on sweets and snacks versus fruits and vegetables did not significantly differ by household income.

### Household food quantities purchased by income

The total ounces purchased for each food and beverage category is shown in Table 3 for home and eating out sources. Overall, the pattern of results was virtually identical to that observed for the per person dollars spent by household income group. Higher income households purchased greater quantities of fruits and vegetables, sweets and snacks (home sources only), eating out entrees and side orders, and non-caloric beverages. Compared with lower income households, higher income households purchased a smaller percent of their total beverage ounces as sugar sweetened beverages and paid more per ounce for fruits and vegetables.

**Table 3:** Per Person Ounces for Food Categories from Home and Eating Out Sources by Income

	Monthly Median Home Ounces Per Person by Income				Monthly Median Eating Out Ounces Per Person by Income			
**Food Category**	**Low**	**Med**	**High**	**P**	**Low**	**Med**	**High**	**P**

Foods								
Fruits/vegetables (includes potatoes)	135	183	212	.003	9	9	18	.003
Sweets/snacks	62	95	94	.011	4	8	9	.173
Prepackaged entrée								
<500 cal	1	1	1	.481	---	---	---	---
>500 cal	17	21	14	.256	---	---	---	---
Eating out entrée	---	---	---	---	39	51	62	.033
Eating out side	---	---	---	---	2	3	5	.029
								
Beverages								
Sugar beverages	76	120	41	.374	21	15	30	.078
Non-caloric Beverages	0	27	216	.001	0	2	10	.001
fruit/vegetable juice	38	32	32	.326	0	0	0	.220
All home (or away) beverages	144	253	385	.051	42	45	105	.002
Eating out alcoholic beverages	----	----	----	----	3	3	8	.006
Eating out specialty drinks	----	----	----	----	3	10	10	.014
Percent of total beverage ounces sugar beverages	44	50	30	.004	----	----	----	----
Price per ounce home fruits/vegetables	.08	.08	.10	.007	----	----	----	----
Per person total ounces fruits/vegetables	146.9	196.63	216.93	.003	----	----	----	----
Per person total ounces sweets/snacks	75.0	110.93	109.64	.029	----	----	----	----
Per person ounces total sugar beverages	113.7	143	124.17	.471	----	----	----	----

## Discussion

It was initially hypothesized that higher income households would spend more on healthful foods and less on sugar-sweetened beverages and snack foods [1-3,5]. The results of the present research show that relative to lower income households, higher income household spent more dollars per person on both healthful and less healthful foods. These findings are consistent with US national consumer expenditure survey 2004-2005 data that show low-income households report food spending of US $413 per month compared with US $870 per month among high-income households [31,32]. Also consistent with US national consumer expenditure survey data, higher income households purchased significantly more fruits and vegetables compared with lower income households. In the 2004-2005 US household consumer expenditure data, higher income households spent US $76 per month and lower income households spent US $49- US $57 per month on fruits and vegetables [31,32]. In the US national consumer expenditure survey 2004-2005, higher income groups purchased significantly more foods classified as "other," which include some of the foods for which higher income households in the present study spent more, such as snacks, sweets, prepackaged foods, frozen meals and nonalcoholic beverages. Data from the present study and the national data suggest that higher income households purchase more fruits and vegetables and discretionary convenience foods compared with lower income households. Only sugar sweetened beverages, when examined as a proportion of home spending, were significantly higher among lower income households. Other data suggest more subtle differences among lower and higher income households in the healthfulness of their food purchases. For example, US individual dietary intake survey data show that lower income households consume significantly more beef compared to higher income households [33]. Other data show that meat is a high priority food item among lower income households, while fruit and vegetables are not [34]. These data are suggestive and make it clear that additional focused research with precise measures of household food spending and detailed measures of specific foods purchased are needed. This type of research will shed light on the process by which lower income households spend less money for perhaps more energy dense foods that may contribute to excess weight gain and obesity [1-3,35]. It also may help explain how higher income households are able to spend more money on food and beverages but remain less likely to develop obesity.

Food purchase sources were also hypothesized to differ by household income, such that lower income households would purchase a larger proportion of their food purchases from sources such as convenience stores and small markets, which typically have more expensive and less healthful food choices. The results of the present study did not support this hypothesis. Lower income households did not differ from higher income households in the per person dollars spent at discount chain grocery stores, small grocery stores, convenience stores, gas stations or nonfood stores. The majority of food purchases were from discount chain grocery stores, regardless of household income level. Higher income households spent significantly more dollars per person from premium chain discount stores, wholesale stores and specialty food stores. Thus, in this sample of households, access to large chain retail food outlets does not seem to be a barrier specific to lower income households.

Higher income households spend a larger proportion of their food dollars eating out [31]. US national consumer expenditure 2004-2005 survey data show that low income households spend 26% of their food dollars eating out, compared with 47% among high income households [31]. The present study found that higher income households spent 37% of their total food dollars eating out, compared with 27% among lower income households, a marginally significant difference. As a proportion of eating out dollars, low income households in the present study spent significantly more on carry-out food places, and higher income households spend significantly more at full-service restaurants. These eating out food source patterns may reflect choices about food cost and food quality that differ by household income and may contribute to the low-income obesity paradox [1-3,35].

These results suggest that the overall amount of money available for food purchases may be the main characteristic related to food purchases by households of different income levels, more than a lack of access to food outlets. Although lower-income households may shop at similar food sources, the types and quality of the foods they purchase may be different from higher income households. For example, in the present study, lower income households may have economized by purchasing less expensive versions of food such as fruits and vegetables. However, it does appear that when they do purchase beverages from stores, a large percent of the beverages are sugar beverages. They also chose carry out restaurants more often than full-service restaurants. Thus, the lower income households appear to have choices about where to shop and what to buy. Higher income households may have more choices, given the greater amount of money they can afford to spend on food of all types from all sources. Food price is less of a barrier to purchase choices for high income households.

### Strengths and Limitations

The present study had several strengths. Detailed data were available on the frequency of food purchases from a wide range of retail food sources over a lengthy time period. The sample was a diverse community sample of households with a range of configurations of adults and children. Eating out sources were included in the receipt collection and annotation. Limitations of the study included the lack of coding of every grocery store food and beverage item purchased and the study's focus on certain targeted food and beverage categories. It is not known whether the receipt collection captured all food and beverage purchases during the four week period, nor whether the purchases of all household members were captured. The number of eating-out receipts declined over the four-week period, suggesting that frequent small purchases from coffee shops or fast food places may not have been completely captured [28]. However, 88% of the household primary shoppers reported that their grocery store food and beverage purchases were well-represented in the receipts data, and 75% reported that their restaurant and fast food purchases were well-represented in the receipt data [28]. The number of receipts differed by household income, which could have biased the observed results in some way.

The households that comprised the sample were not representative of the community and self-selected to participate in a weight gain prevention intervention involving eating, physical activity and television viewing behaviors. Compared to national census statistics, the sample was comprised of more married (65% versus 51% nationally), well educated (26% greater than college degree versus 9% nationally) and higher income people (65% income > = US $50,000 versus 50% nationally) [36]. Thus, the generalizability of the results is not known.

Future research is needed to examine household income-related food and beverage purchasing patterns in a representative sample of households. Documentation of food sources other than retail food stores is needed, particularly among lower income households that may receive food from sources such as food shelves or federal food programs. Examination of the full range of foods purchased will also provide more complete data about household income differences in food purchases, both sources and types of foods and beverages. A more detailed collection and analysis of specific food types and prices paid will help provide a more clear understanding of how lower income households spend less money for food but perhaps purchase more calories and thus increase risk of excess weight gain and obesity. These data will also help clarify how higher income households can spend more money on food and beverages but remain at lower risk of excess weight gain.

## Conclusions

Higher income households spent more money on all types of foods from a wide range of sources. Lower income households spent a larger proportion of their eating out dollars at carry out places, and a larger proportion of their home beverage purchases were sugar sweetened beverages. More detailed research on household food spending can promote a better understanding of how retail food sources, food prices and specific foods purchased contribute to household energy purchased, energy balance and risk for excess weight gain.

## Competing interests

The authors declare that they have no competing interests.

## Authors' contributions

SAF conceptualized the research idea and drafted the manuscript. NRM and MW conducted data analysis, provided scientific input and edited the manuscript. All authors read and approved the final manuscript.
